# Treatment of refractory esophageal stenosis after endoscopic submucosal dissection with magnetic compression anastomosis

**DOI:** 10.1055/a-2279-6910

**Published:** 2024-03-20

**Authors:** Miaomiao Zhang, Huanchen Sha, Guifang Lu, Hairong Xue, Yi Lv, Xiaopeng Yan

**Affiliations:** 1 162798 Department of Hepatobiliary Surgery, The First Affiliated Hospital of Xiʼan Jiaotong University, Xiʼan, China; 2 162798 Shaanxi Provincial Key Laboratory of Magnetic Medicine, The First Affiliated Hospital of Xiʼan Jiaotong University, Xiʼan, China; 3 Department of Gastroenterology, The First Affiliated Hospital of Xiʼan Jiaotong University, Xiʼan, China

Magnetic compression anastomosis (MCA) has been previously used for the treatment of colorectal stenosis 1 2 and pediatric esophageal stenosis or atresia 3 4 . However, there have been no reports of MCA being used for the treatment of esophageal stricture after endoscopic submucosal dissection (ESD) in adults.

A 73-year-old man underwent ESD for early esophageal cancer and experienced dysphagia 1 month after the procedure. Gastroscopy revealed esophageal stenosis, for which he underwent three sessions of balloon dilation and one session of esophageal stent placement. Unfortunately, the esophageal stenosis continued to worsen, as confirmed by esophagography and gastroscopy ( Fig. 1 ).

**Fig. 1:**
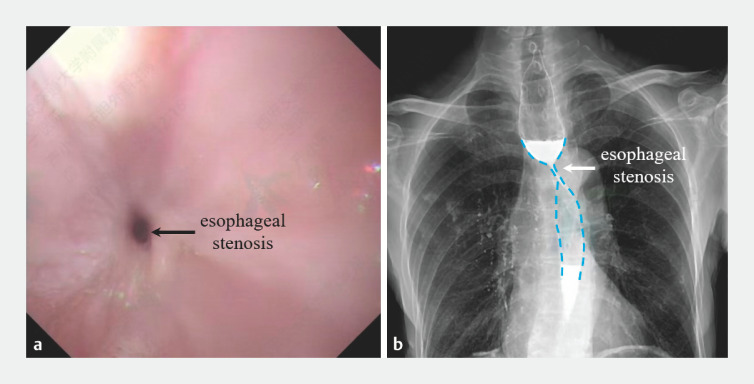
Persistent esophageal stenosis following endoscopic submucosal dissection: **a** gastroscopic image; **b** esophagogram.

The patient declined esophagectomy for the stenosis, and therefore MCA was recommended. A schematic diagram illustrating the surgical planning and the magnets is shown in Fig. 2 . Following anesthesia, the patient underwent laparoscopic gastrostomy, and the proximal end of the esophageal stenosis was reached through oral endoscopy. After multiple attempts, the zebra guidewire was successfully passed through the stenosis to enter the stomach. From the stomach, the guidewire was pulled out of the abdominal cavity. Then, the parent magnet and the gastric tube on which it sat were inserted over the guidewire and sent to the stomach. The gastric tube was pulled out orally through the stenotic segment. The daughter magnet was then passed over the head of the tube and pushed by the gastroscope towards the proximal (oral) end of the esophageal stenosis. The daughter and parent magnets were attracted together ( Fig. 3 ; Video 1 ).

**Fig. 2:**
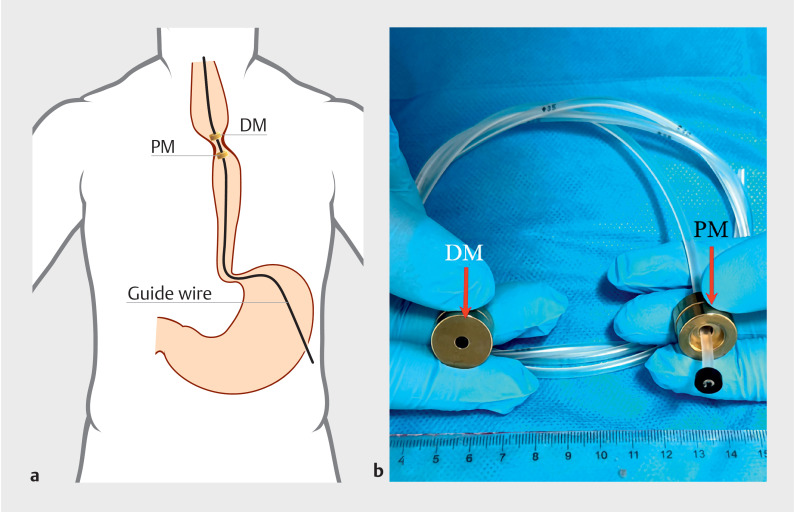
Surgical planning for magnetic compression anastomosis: **a** the daughter magnet (DM) and the parent magnet (PM) were inserted through the mouth and gastrostomy respectively; **b** parent and daughter magnets

**Fig. 3:**
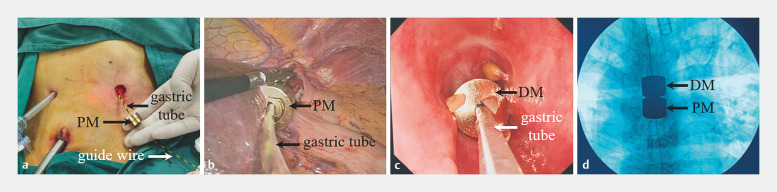
Surgical procedure: **a** , **b** the parent magnet was pushed into place; **c** the daughter magnet was pushed into place under gastroscopy; **d** the two magnets were attracted together.

Surgical procedure for magnetic compression anastomosis to treat refractory esophageal stenosis following endoscopic submucosal dissection.Video 1

The magnets were removed endoscopically, and 11 days after surgery an esophageal stent was inserted ( Fig. 4 **a** , **b** ). After 3 months, the stent was removed ( Fig. 4 **c** , **d** ). The patient has been followed up for 8 months and has not received any further endoscopic treatment. He is now able to eat normally. MCA is a potential treatment option for esophageal strictures that do not improve with repeated balloon dilations.

**Fig. 4:**
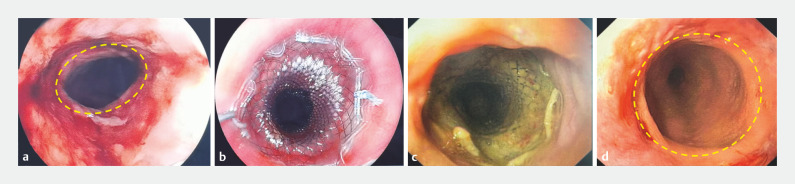
Establishment of a magnetic anastomosis: **a** the magnets were removed 11 days after surgery; **b** an esophageal stent was implanted; **c** , **d** after 3 months the esophageal stent was removed.

Endoscopy_UCTN_Code_TTT_1AO_2AH

## Notes

Endoscopy E-Videos https://eref.thieme.de/e-videos
*E-Videos* is an open access online section of the journal *Endoscopy* , reporting on interesting cases and new techniques in gastroenterological endoscopy. All papers include a high-quality video and are published with a Creative Commons CC-BY license. Endoscopy E-Videos qualify for HINARI discounts and waivers and eligibility is automatically checked during the submission process. We grant 100% waivers to articles whose corresponding authors are based in Group A countries and 50% waivers to those who are based in Group B countries as classified by Research4Life (see: https://www.research4life.org/access/eligibility/ ). This section has its own submission website at https://mc.manuscriptcentral.com/e-videos .
